# Trauma of separation: the social and emotional impact of institutionalization on children in a post-soviet country

**DOI:** 10.1186/s12889-023-15275-w

**Published:** 2023-02-20

**Authors:** Leyla Ismayilova, Emily Claypool, Emma Heidorn

**Affiliations:** The University of Chicago, Crown Family School of Social Work, Policy, and Practice, Chicago, IL USA

**Keywords:** Complex developmental trauma, Orphanage, Institutional care, Child mental health, Post-soviet

## Abstract

**Background:**

In the former Soviet Union (fSU) region, which has the highest rate of institutional care worldwide, ‘*social orphans’* —indigent children who have one or both parents living—are placed in publicly run residential institutions to receive education, food, and shelter. Few studies have focused on understanding the emotional effects of separation and life in an institutional environment on children who grow up with their families.

**Methods:**

Semi-structured qualitative interviews (*N* = 47) were conducted with 8- to 16-year-old children with a history of institutional care placement and their parents in Azerbaijan. Semi-structured qualitative interviews were conducted with 8- to 16-year-old children (*n* = 21) involved in the institutional care system in Azerbaijan and their caregivers (*n* = 26). Trained interviewers collected narratives about children’s experiences prior to being separated from their families while living in an institution, as well as the impact of institutional placement on their emotional well-being. We applied thematic analysis with inductive coding.

**Results:**

Most of the children entered institutions around the school entry age. Prior to entering institutions, children had already experienced disruptions within their family environments and multiple traumatic events, including witnessing domestic violence, parental divorce, and parental substance abuse. Once institutionalized, these children may have had their mental health further impaired by a sense of abandonment, a strictly regimented life, and insufficiencies of freedom, privacy, developmentally stimulating experiences, and, at times, safety.

**Conclusion:**

This study illustrates the emotional and behavioral consequences of institutional placement and the need to address accumulated chronic and complex traumatic experiences that occurred before and during institutional placement, which may affect emotion regulation and the familial and social relationships of children who lived in institutions in a post-Soviet country. The study identified mental health issues that could be addressed during the deinstitutionalization and family reintegration process to improve emotional well-being and restore family relationships.

## Introduction

Countries of the former Soviet Union (fSU) and Eastern Europe have the highest rate of child institutional care worldwide [1]. As these regions undergo deinstitutionalization reforms [2], understanding the effects of institutional placement on children’s mental health functioning is critical as children transition from residential institutions into family-based or community-based settings.

Studies on children adopted from orphanages in Eastern Europe show that institutionalization has a strong negative effect on developmental outcomes and that children with a history of institutionalization are more likely to exhibit emotional, behavioral, and relational problems (e.g., severe antisocial behavior and aggression towards others, disturbances of attachment) [3–6]. Following multiple traumatic experiences in institutions, including physical or sexual abuse [7], some children exhibit posttraumatic stress symptoms and self-harming behaviors and report depressive symptoms [8–10]. Children who grow up in institutions also have limited life opportunities as adults, struggle to adjust to society, and are more likely to develop mental illness or substance use addiction [11, 12].

While a substantial body of research has examined the impact of institutional placement on child mental health, the existing literature is primarily focused on Eastern European countries (e.g., Bulgaria, Romania, Russia, Ukraine) [13–17]. While post-Soviet countries had a similar centralized system of institutional child care, there are major socio-cultural and political-economic differences that affect patterns of institutional placement, and limited information is available regarding children who grew up in institutions in Post-Soviet countries in the Caucasus or Central Asia [18–20]. Furthermore, many of the available studies focus on children who were placed in institutions at birth and had no contact with their biological parents [21–23], or were adopted from institutions internationally [24–27]. A significant number of children in institutions in Azerbaijan and its neighboring countries have at least one living parent and were placed there by parents after infancy, often close to school entry age, primarily to obtain free public services (such as education, food, and shelter) and—as a consequence of gendered poverty—to relieve financial strain on the family [28].

Societies in the Caucasus or Central Asia are characterized by collective cultural norms and familial practices, where a woman’s role is strongly linked to taking care of the family and child rearing [29]. The majority of mothers who placed their children in institutions in Azerbaijan identified single parenthood following a divorce or loss of a breadwinner as a precipitating factor to the economic and emotional hardships they faced [30]. Furthermore, unlike the children placed in institutions since infancy, children placed at an older age are able to reflect on their experiences of family separation. This provides an opportunity to elicit qualitative accounts of their experiences. Many children also maintain contact with their families, visiting their homes during school breaks or holidays [30, 31], and may have varied experiences with institutional placement.

Due to the very young ages of the child participants and the limitations of predominantly quantitative methodology, previous studies have not examined the perspectives of children from these institutions or the sociocultural context of their experiences. Through children’s and caregivers’ reflections on family separation, this qualitative study aims to explore (1) the traumatic experiences that affect the emotional well-being of children with a history of institutionalization and (2) the role of the institutional environment in shaping children’s emotional well-being.

Deinstitutionalization reforms in the fSU region focus primarily on administrative restructuring of the child care system and replacing large-scale residential institutions with family-based and community-based alternatives [19]. In Azerbaijan, specifically, the total number of institutions, primarily boarding schools, as well as the total number children placed in institutions has reduced following the reforms [32]. The gatekeeping, foster care and adoption services have been transformed and digitalized to make the process more efficient and transparent. In addition to preventing new children from entering into the system, the reforms are currently focused on local adoption and foster care until adoption for children deprived of parental care.

Less attention has been paid to strategies and interventions focused specifically towards restoring the mental health functioning of children, who have left the institutional care system and especially those, who returned to their families [18, 33, 34]. This study was conducted during a formative phase to explore the mental health needs of children reunifying with their biological families to inform the development and adaptation of an intervention designed to improve their mental health wellbeing. This study’s findings have the potential to inform other interventions, policies, and services that could improve the psychosocial adjustment of children with a history of institutional placement.

The theoretical framework of Attachment theory—which deals with how a child’s early formation of bonds with caregivers affects the development of relationships and emotional well-being at a later age [35]—informs this study’s understanding of a child’s development, attachments, and social-emotional well-being during reunification. The quality of attachments beyond early childhood are also critical to consider, such as the impact of caregiving practices on child and adolescent well-being, as well as the importance of attachments with other family members and peers [36]. Theories of attachment offer a model for understanding how children respond emotionally to separation from caregivers during institutionalization, and how this disruption in attachment might impact the child–caregiver relationship during reunification.

As this study highlights, children who have spent time in institutions have experienced a series of traumatic events, from early childhood through adolescence. Complex developmental trauma theory offers a framework through which to understand the long-lasting impact of these multiple continuing traumas on the development and social-emotional well-being of children [37]. System-induced traumas—such as those related to living in the harsh conditions of an institution—are also critical to consider in order to understand the impact of complex traumas [38]. Theories of development suggest that children go through a series of developmental stages and acquire new skills and competencies at each stage to help them adapt to new environments and face challenges. Disruptions in this developmental trajectory such as placement in institutions and isolation from caregivers may result in social-emotional difficulties, especially in emotion regulation [39, 40]. The traumas and challenges a child has experienced while in an institution might impact the ways in which that child manages feelings of anger or anxiety, as well as aggressive or internalizing behaviors.

## Methods

This study warranted qualitative phenomenological methodology to explore the children’s and caregivers’ personal experiences prior to and during family separation. The study protocol was approved by the University of Chicago Crown Family School of Social Work, Policy, and Practice and Chapin Hall Institutional Review Board (IRB15-0051) and by the Ethical Committee at the Azerbaijan Medical Association / AzMA (Approval #34/01).

### Study setting, participants, and sampling procedure

The study targeted children with a history of institutional placement in public orphanages, *internats* (similar to boarding schools), or alternative care facilities (e.g., group homes, SOS Children’s Villages) with at least one surviving biological parent or close relative. Orphanages and ‘internats’ in Azerbaijan are public and under the auspices of the Ministry of Education (with the exception of institutions for children with special needs that were not included in this study). A caregiver was eligible to participate in the study if he/she met the following inclusion criteria: [1] was a biological parent or kin relative (e.g., grandparent, aunt) who served as the primary caregiver; [2] was at least 18 years old; [41] had a child currently living in an institution or had recently reunited with a child who lived in an institution (orphanage or internat); and [3] this child was between the ages of 8–16. The study utilized convenience sampling and recruited children ages 8–16 with a history of institutionalization (*n* = 21) and their parents or primary caregivers (*n* = 26). Among caregivers, 15 were the parents of children currently living in institutions, and 11 had already taken their children back from institutions. No eligible participants refused to participate in the study.

We approached seven residential institutions in two cities of Azerbaijan (Baku and Quba). Administrators from the selected institutions identified caregivers who were potential participants, including those who had recently reunited with their children, and informed them of the opportunity to participate in the study. The project coordinator then contacted these caregivers to provide additional information about the study, informed them that study participation was voluntary, and conducted informed consent procedures and screening interviews to determine their eligibility. Children underwent the assent process separately from parents to avoid coercion.

### Data collection

The semi-structured face-to-face interviews collected qualitative information about children’s and parents’ life experiences before and during institutionalization. Interviews were conducted in the Azerbaijani language in a secure and private location at participants’ homes (after reunification or during children’s home visits). The interviews took approximately 30 min with the children and 60 min with the parents.

Interviews were conducted between 2015 and 2016 by two local female interviewers who were trained master’s-level social workers and had experience working with children in the institutional care system. The study’s principal investigator from the University of Chicago, who also has clinical experience of working with children from institutions in Azerbaijan, provided the interviewers with additional training on interviewing skills, the ethical conduct of research, and reporting and managing adverse events.

Two separate interview guides for children and caregivers were designed in order to inform the intervention strategies aiming to improve the well-being of deinstitutionalized children. The questions were guided by Attachment and Emotional Regulation theories identifying areas potentially affected by family separation. The interview guides included five sections and focused on the period prior to institutional placement (e.g., *How did you separate from your child? What circumstances led to your separation from your child? How was the decision made to place this child in the institution and not other children?*), during institutional placement (*Could you tell me about your life at the orphanage?*), the period of reunification or barriers to reunification (e.g., *What do you think about taking your child back from the orphanage? What could help you reunite with your child or make it easier for you to take care of your child when he/she is back to live with you? Are there things that worry, scary or bother you about going back to live with your family?*), and program development (e.g., *What kind of assistance would be helpful to make your family life more stable and make it easier to care for your children? If there was a program, what would be helpful for you to learn to help you with your child and help you keep your family together? What can help you achieve your dreams?).*

To explore mental health needs prior to and after institutional placement, in this manuscript, we focused on the following questions for caregivers from the interview guide: *What do you know about your child’s life at the orphanage? How do people treat him/her there? What types of changes has your child experienced after leaving home? How has living in the institution affected or changed your child? How does your child react when you or other family members visit your child at the institution?* Questions from the interview guide for children analyzed in this manuscript included: *Could you tell me about your family? What do you like / did not like about living in the orphanage? When and how did you come here? How do you spend your time here? Who are your friends? How do other people treat you in the orphanage? In what situations do you feel especially happy / sad or lonely / angry / scared? What do you do in such situations? With who do you talk to or share how you feel? How do your parents or other family members react when you are very sad / angry / scared?*

The findings from other sections exploring the reasons for institutionalization, motivation and potential obstacles for family reunification, and children’s and caregivers’ experiences following the deinstitutionalization are described in separate manuscripts [30, 42] and have informed the design and adaption of the multi-level mental health, family strengthening and economic intervention model currently undergoing testing in a randomized control trial [43].

### Data analysis

Audio-recorded interviews were transcribed verbatim and subjected to random quality assurance checks that compared sections of audiotapes to transcripts. Transcripts were then translated into English. Four coders (two in Azerbaijani and two in English) conducted thematic analysis [44, 45] using Dedoose, a web-based software for the analysis of qualitative data [46]. Data were first assessed using open coding to generate overarching themes and patterns, followed by focused coding in later cycles [47]. Coders analyzed data by reading transcripts, listing themes and concepts for the subsequent analysis, and organizing, reducing, and coding data. Portions of the text were selected and organized thematically by what we discerned as mental health needs as well as traumatic experiences prior to and during institutionalization. A set of analytic coding categories was created via axial and selective coding and revised through a process of contrast and comparison, and discrepancies were discussed and resolved. The interviews were coded and analyzed to explore the following a priori domains: [1] *children’s exposure to trauma and family separation;* [2] *the range and types of reactions to separation and traumatic events; and* [41] *how children deal with trauma, separation, and deprivation.*

## Results

**Table 1:** Sociodemographic characteristics of the sample

CHILD CHARACTERISTICS (*n* = 21)	Mean (*SD*) / Frequency (%)
**Child’s age (in years)**	*M* = 12.9 (*SD* = 2.7) (min 8 - max 16 years)
**Gender** Boys Girls	13 (61.9%) 8 (38.1%)
**PLACEMENT CHARACTERICS**	
**Child’s age at institutionalization (in years)**	*M* = 6.8 (SD = 2.3) (min 3 – max 13 years)
**Type of institutional care setting**: Orphanage or internat (‘boarding school’) Alternative care (e.g., small group home)	16 (76.2%) 5 (23.8%)
**Child’s reunification status**: Reunited with their caregiver Currently in institution	11 (52.4%) 10 (47.6%)
**Length of institutionalization (in years)**	*M* = 5.4 (*SD* = 3.2) (min 1 – max 11 years)
**CAREGIVER CHARACTERISTICS (*n*** **= 26)**	
**Gender (female)**	26 (100%)
**Primary caregiver type**: Biological mother Grandmother	24 (83.3%) 2 (9.5%)
**Caregiver’s age (in years)**	*M* = 40.6 (*SD* = 8.3) (min 27 - max 60 years)
**Marital status**: never married currently married divorced widowed	2 (7.7%) 2 (7.7%) 18 (69.2%) 4 (15.4%)
**Education**: Primary school grades (grades 1–4) General secondary education (grades 5–9) Complete secondary school (10–11) Vocational school (college, technikum) Higher education (university degree or higher)	3 (11.5%) 6 (23.1%) 11 (42.3%) 6 (23.1%) 0 (0%)
**Employment status**: not employed employed retired	8 (30.8%) 16 (61.5%) 2 (7.7%)

## Sample socio-demographic characteristics

As presented in Table 1, the child participants were between the ages of 8 and 16 (*M* = 12.9, *SD* = 2.7); 62% were boys. The majority of these children (*n* = 16, 76.2%) were placed in public orphanages or *internats* (boarding schools), and five children (23.8%) had a history of placement in alternative care settings (e.g., group homes). Most institutional placements took place primarily around the child’s school entrance age (*M* = 6.8, *SD* = 2.3), and children spent over five years on average in institutions. A third of the child participants were in institutions along with their siblings.

Although the inclusion criteria for caregivers were not limited by gender, all interested and eligible caregivers were female (24 were biological mothers and two were grandmothers). Two mothers were currently married, and the remaining mother caregivers (85%) were single and were parenting alone (as a result of divorce or the death of their husbands). Over one-third of the female caregivers, all of them biological mothers, did not complete secondary education. Approximately two-thirds of the caregivers reported being employed, often in low-wage jobs (e.g., dishwashers, cleaners).

## Qualitative findings

### I. Traumatic experiences prior to institutionalization

Many children had already been exposed to a number of traumatic experiences prior to entering an institution (Table 2). These traumatic experiences included witnessing domestic violence as well as experiencing child abuse, often associated with the father’s substance abuse. Children in the study also experienced multiple losses, including the dissolution of the family unit and growing up without a father.

**Table 2:** Qualitative themes and sub-themes associated with poor child mental health

*Thematic Category*	*Sub-Themes*
**Traumatic experiences prior to institutional placement**	Witnessing domestic violence Father’s substance use Parental separation or divorce Absentee father
**Traumatic experiences during institutional placement**	Sense of abandonment Cognitive ambivalence Silent resentment Maltreatment in institution
**Institutional Environment**	Limited privacy (Crowded space) Limited stimulating environment (Deprivation) Limited personal freedom (Strict regime / discipline) Limited safety (Maltreatment / bullying)

### Ia. Witnessing domestic violence

Exposure to domestic violence—typically committed by the father prior to institutionalization—was a very common theme among the children and female caregivers interviewed. A mother of three described how her ex-husband was often brutally violent towards her and their children:

After marriage, I found out that my husband had some problems—he was a drug user. I couldn’t notice it from the beginning as nobody drank or gambled in my family. He was beating me every day and my son Samir used to stand in front of me saying, Beat me, but don’t beat mom. This way my children’s nerves got damaged. I decided to split up for my children, not for myself.

Most mothers reported that the violence witnessed and experienced by children at home resulted in emotional and behavioral difficulties and served as a catalyst for separation. A mother of three described how the brutality she and her eldest child faced started to have an impact on her son’s personality:

My husband is my cousin from my mother’s side, and he married me because his mother [my aunt] insisted. After her death, he left the family and returned 6 months later, and Oguz [my son] witnessed all these problems. Then, my husband had problems getting along with my son, and he hurt him and kicked out of the house. After all this, I saw that my son turned out to be colder in nature, less interested in everything. For example, he did not care what he had to wear, whether it was clean or dirty or torn. What I said to him inside [the house], he used to forget outside.

Mothers expressed concern that their children became ‘*introverted*’, disinterested, and emotionally ‘*cold*’ after witnessing domestic violence, while also exhibiting fearful behaviors. A mother, whose 13-year daughter currently lives in an institution, recounted:

She was very scared of her father, and when he would come home, she used to hide under the bed and say, I don’t want to come out.

### Ib. Paternal substance abuse

The domestic violence experienced by children and their mothers was often coupled with—or precipitated by—paternal substance abuse. The same mother detailed the violence she and her daughter suffered, which ultimately led to the separation from her husband and, eventually, the institutional placement of her only child at the age of 5:

After marriage, I was there for 3 years, and those three years of my life were torture. My husband used to drink and smoke weed and beat me at home. Once he beat my daughter awfully and that night I took my child and left home.

Realizing the negative impact on children, mothers explained that they wanted to protect their children and provide a better environment by placing them in an institution. A mother of a 10-year-old boy described the following:

My husband was an alcohol user. He used to take [our son] with him out to cafes, and I saw that my son’s behavior changed. Frankly, that was also a reason to place [my son] in the institution so that he could be distant from his father and not to be involved in those things…. My sister used to take my son to a psychologist to learn the reasons for changes in his behavior. [My son] was getting angry on his own, without a reason and reacted to every tiny thing. I was really scared about my child’s future. They explained that the reason is rooted in the family, as his father is using alcohol.

### Ic. Living through parental divorce and family dissolution

Many children experienced the divorce and dissolution of the family unit prior to entering an institution, which caused a great deal of distress for the children, especially given a cultural environment where divorces are socially condemned and single motherhood is heavily stigmatized. One mother shared her teenage son’s negative reaction to her separation from his father:

When we were in the process of separation, my son witnessed everything. Therefore, I noticed that he was going in the wrong direction and getting lost. We had reached such a moment that I started to feel that he didn’t want me or even his brother and sister... He was all about himself. He said that he did not want to see his father. He even asked his mother in [the family group home] to tell me not to agree that he meets with me because he was too subjected to psychological pressures.

### Id. Paternal disengagement and the perceived stigma of being raised without a father

For many children in the study, their father’s separation from the family unit prior to institutional placement was the first experience of abandonment that caused a significant disruption in the child-parent relationship. As illustrated by one mother, her son’s reaction to not having a father in his life was also influenced by cultural stigma against single-parent households:

My son visits relatives and sees other children in their family, how they live. He does not have a father, with his single mother. Sometimes I cannot buy what he wants, and then he questions, “Why did my father act like that?” I do not know, he is a child…but grew up. Why he left us, why other fathers are beside him. He sees that his father is an alcohol addict and his father is with him.

The stigmatized identity arises from the cultural belief that a child, especially a boy, must have a father figure to learn proper behavior and be disciplined appropriately. Some mothers showed concern that their sons, in particular, were having emotional and behavioral problems because they grew up without a father figure. It was often the case that mothers faced significant stigma for raising their children alone after separating from their husbands. This mother reports facing rejection from her own family following divorce:

I have gone through tough times to keep my daughter with me. My sisters and brothers left me outside with my small child in my arms. Her father also didn’t want her. It was all their fault. They always kept saying, “Get married, get married,” and once I didn’t have a successful family, I was supposed not to return back to the family. Even if a woman returns back [to her family], she has to leave her baby, but I didn’t do it.

### II. Effects of institutional placement

#### IIa. Trauma of separation

The moment of separation during the institutional placement was traumatic for all children as well as their parents. Most children described sadness and yearning to be with their parents; the separation and moments of visitation were often tearful. A mother of a teenage boy who has been living in an institution since second grade described the following:

Usually he misses me and I visit him…every ten days. He misses a lot. When he was younger, he used to cry at night a lot. He used to take my handkerchief and scarf to put under his pillow at night and fall asleep by smelling it. He used to cry a lot when he was little, now also, but I had to leave him here as I do not have a home. [When I visit him], he smells me so much, saying, ‘Mommy, mommy’. He is an introverted child. I cry a lot when I visit him and he asks me not to. He says that when I get out of here, I will take care of you. He notices how touched I feel every time I see him. He hardly refrains himself from crying with me.

The age a child was placed in the institution proved to be an important factor in how children experienced and responded to separation from their families. The average age of a child’s placement in an institution was approximately 6–7 years old. These children had experienced life at home and had built relationships with family and caregivers prior to being placed in an institution. One mother expressed why she felt her children could not adjust to the life in the institution:

They have been living there for 4 years, and the psychologist, director and other staff there say that these children cannot adjust here; they cannot accept it because they were big already, when they were placed there and they came from home. If you talk to them or ask staff there, you will see that they are distinct from other children. Even at school, they say that these children do not seem to come from [the institution], they are very different. When sponsors visit [the institution] to bring some gifts, my daughters do not go out to meet them, do not accept gifts, and do not let them take their photos, but the staff insists that they join all other children. Everybody is surprised why they are different. I understand them because children living in the orphanage from birth are different from those coming from families. I do not wish any mother to separate from their children and children to stay apart from their parents.

**Ambivalence and Silent Resentment.** None of the interviewed children reported being prepared for or properly informed about their institutional placement. Older children attempted to grapple with or justify their parents’ actions, sometimes reporting that it was done to provide better educational opportunities. For example, a 12-year-old girl living in an institution stated:

*Everybody wants good for their children. My parents did not bring me here of their own free will—they knew that here I would be taken better care of, that is why they placed me here. I cannot tell you what I do not know* [referring to that she was never told about the true reasons for placement]. *Maybe they wanted me to have an education, to be a smart girl, learn some skills and have my own students… Inshallah, it will happen! I want to be a piano teacher.*

Younger children often wavered between the feeling of resentment around being abandoned by their caregivers and missing their parents acutely. Children sometimes blamed their parents for institutionalization (although not very openly), describing their situation as akin to abandonment. One mother, whose two sons are still in an institution, described the following:

They blamed me [when they were placed in the institution] that I was a bad mother and abandoned them here. But now they have changed a little bit, maybe because they are growing up.

Children shared that after spending years in the institution, they eventually learned to accept their parents’ financial precarity and stated a desire to do their best for their children. A 15-year-old who returned home after living in an institution for 11 years shared the following:

Everything was good, but nothing can replace mother, how much good it can be. Before I was a child and could not understand everything. But after growing up, at [the age of 12–13], I started to understand life. This is life, and such difficulties happen in life. Then, we understood each other. I stayed here because there was no other choice.

Younger children particularly struggled to reconcile the sense of abandonment while trying to understand their parents’ difficult decision. A mother of 9- and 12-year-old girls placed in the institution over two years prior to being interviewed shared that her younger daughter had more difficulties handling the separation, which resulted in a more detached attitude:

My elder daughter cries when she sees me, but the younger one does not care. When they are home, the younger one says that at home we do not have the good conditions that they have in the internat, it is good there and I want to go and stay there.

While many parents maintained contact with their children and continued visiting their children in the institution, the frequency of visits depended on the parents’ personal and socioeconomic circumstances (e.g., distance from the institution, costs of travelling) as well as the type of institution (e.g., very strict visitation rules and no contact via phone in alternative care institutions run by NGOs, compared to public institutions). Some children went home every weekend or on holidays. One mother reported visiting her child every Wednesday to give him a bath. A handful of children said their caregivers visit very rarely, at times only once per month or per year. Naturally, the frequency with which parents visited their children affected the children’s sense of support, and more frequent visits helped to maintain the parent–child bond. However, such visits were not always easy for some children to handle, as they demonstrated highly ambivalent emotional responses to their parents. A mother of a 12-year-old girl who had lived in the institution since the age of 7 recounted the following:

*When I visit her twice a week, I notice that she gets used to it. When I visit her once a week, I see that she turns cold to me. I am friendly and kind with her; when I visit her, I hug and kiss her, but she says, “Mom, don’t hug me”. I miss her, and I see that she became cold with me. It feels like she gets used to me, while I am there and when I leave, she misses me.*

Some children who had experienced multiple disruptions in caregiving arrangements reported numbness or flat affect:

I was 5–6 years old back then, I remember that [my mother] used to leave me with my aunt. Small children always cry or miss when they separate from their mothers, but somehow I never had that feeling, nothing. It is me who has a problem.

#### IIb. Difficulties in emotion regulation

Anger and aggression were commonly reported externalizing problems for children during and after their time in the institution. This anger and aggression were often a result of traumatic experiences pre-institutionalization, resentment during separation, and adjustment to reunification.

One mother reported aggressive behavior in her children prior to placing them in an institution. The children’s home life had been unstable and occasionally unsafe. The mother and children experienced severe violence and abuse from the father. The mother was sent to the hospital due to injuries from the father’s abuse. Another mother reported a similar experience with a change in behavior in her 10-year-old son, who had been living in the institution for the past two years.

*“My son was getting angry on his own, without a reason and reacted to every tiny thing. I was really scared about my child’s future. He was getting out of control and Icould not manage him. I thought he could join other children. My sister used to take him to a psychologist to learn the reason for the changes he used to have. They explained that the reason is rooted in the family, as his father uses alcohol.”*

Both parents and children reported that children faced difficulties in managing impulses and emotions, particularly in terms of managing anger and other distressing emotions. A 15-year-old girl who had been living in the institution for almost 9 years described struggling to manage seemingly mundane tasks:

*I am hot-tempered, but I like calm and quiet places. I lose my temper very easily over a simple word, anything can irritate me and frustrate me easily… every word. For example, when I read a book, I can’t read the same thing twice, as I don’t have patience and I get frustrated*.

#### IIc. Institutional environment

Children reported a range of experiences in the institution. It was common for children to express that they had no problems there. Many children and caregivers reported feeling supported by the staff of the institution, including teachers, administrators, and psychologists. Some children appreciated having regular meals, a bed to sleep in, and a chance at an education. Two children mentioned that the institution provided a sense of stability and relief from the chaos and instability at home. One child reported liking the institution because she received regular meals on time, and although she missed her mother, she did not want to return home. Nevertheless, with a few minor exceptions, most children reported that they would prefer to be home with their families.

Although institutions did provide access to education for children, the environment in institutions was not conducive to children’s healthy psychological and social development for a number of reasons. Many children realized this as they got older. As one teenage girl noted:

*It is better to be in the internat rather than living on the street. But your morals get violated… The internat is a place that breaks your life...it changes you… So many things happen in front of your eyes…. Even teachers sometimes act improperly in front of your eyes, everybody does...from teachers to children. If you get there as a child, it will 1000% change you*.

### Deprivation and insufficiently stimulating environment

A common negative report about life in institutions was a lack of a stimulating environment. This *‘boredom’*, as children often described it, largely stemmed from the monotony of life and the regimented structure of the institution. When asked to describe daily life in the institution, one female teenager said, *“It was like a movie, the same movie every day, kind of, and it was like repeatedly playing every day…even the food is the same.”* The same teenager reported a low quality of education in the school, which also contributed to her boredom.

I used to sit quietly because I was the only girl, and the rest were boys. Classes were low quality, and I had a dispute with our biology teacher. I wanted to be a doctor, and I was asking him to teach us biology, but the teacher didn’t…. It was not the teacher’s fault, boys in the class used to make noise and not listen to him.

Another child, who had been reunified with his family a few months prior to the interview, said that there was simply nothing to do in the institution. *“When I am [at home], I go for sports, I am not bored. But there in the institution we sit all day thinking what to do; only playing games is boring.”*

### Social isolation

It was very common for children to express loneliness and sadness as a result of their separation from their family. One child said, *“The main problem is me. There are many children here, but I always feel alone and lonely.”* Another child revealed what he called the suffering of other children at the institution and how children would—at times—treat each other poorly and even abuse one another, so that many children felt alone:

Children see a lot of things there, and they suffer, miss their homes. I don’t miss mine too much, but there are children there who miss their family, some even cry.

When a reunited 15-year-old girl was asked to reflect on anything she disliked in the institution, she said:

I could not see my parents, and it makes us feel sad when we do not see them. If we do not have them, why do we need all the nice things there? The only thing I did not like [at the institution] is being separated from my parents. We had everything, except them.

### Undermined safety

Some children and caregivers reported a lack of safety within the institution, including violence between children. This lack of safety resulted in a decreased level of trust in the institution, as well as a higher level of distress and discomfort among children.

*Bullying.* The reports of violence between children involved fighting and bullying from older children. Children reported being made fun of and being beaten up by classmates. This violence was often met with little reprimand or intervention by institution staff members, forcing some children to mediate fights between classmates. A 14-year boy who still lives in the institution reported:

Sometimes they fight with each other; the weak one is beaten by the strong one. When I see, I do not let them fight. Big children also fight, hurt each other, but they cannot tell anybody; they feel scared that somebody can call them a ‘traitor’. Now that I will upgrade to adult class this year, I will not let such things happen.

He also had a message for parents:

First, they should not send their children to the institution for school is not a good place there. It is like here at home I didn’t know many things, but going to school there I learned about and understood a lot of things. My advice to parents is that they should not send their children to the institutions. Children see a lot of things there, and they suffer, miss their homes. I do not miss too much, but there are children there who miss their family, some even cry.

### Limited personal freedom

The strict regimen and discipline carried out in the institutions created an atmosphere where children had little freedom to do as they wanted, a stark contrast to many of their experiences at home. While children primarily complained about the strictly regimented life, parents tried to understand or justify that the strict regimen is necessary because institutions are crowded and under-resourced. A mother of a teenager who had lived in an institution since 2nd grade explained:

He misses many things and feels bored a lot. At home, he is independent: he goes to the market, goes to the bazaar, goes to play outside in the street. But there are rules in the Internat: what time to sleep, what time to wake up, to eat.... It would be difficult to control all these children if they also give them independence in the internat.

A handful of caregivers noted differences in their children’s behavior due to this strict discipline and described feelings of confusion over the contrast of discipline between home and institution. One mother, whose daughter remains in the institution, described the rigid nature of the institution.

It is a closed institution; she cannot dress, eat, or go out as she wants. She goes only to school and back. It is like a prison regime. When she is at home, she does not want to go out to meet neighbors or go to shop. I do not say it is bad there, but too strict there.

### Limited privacy or personal space

In larger institutions, overcrowding contributes to a lack of privacy among children. While overwhelmed by emotionally charged experiences, children in institutions struggle to find a safe and secure foundation from which to effectively cope with intense emotions. In many children, this created a longing for quiet spaces where they could be alone. A 15-year-old girl who had been living in the institution for the past 9 years described her constant yearning for inner peace:

*Our [staff] psychologist asks us to draw pictures to test our psychological condition. She asks me why I always use such dark and dull colors. She tells me to think about funny things; she makes us write stories about places we would like to be. I always write that I want to be in a quiet forest, and she asks whether I have any other wish. She says other children write they want to be in other countries, in amusement centers, but you always write about a quiet forest*.

Children described feeling a sense of comfort when being alone, in contrast to the chaos they may find at the institution as well as at home. A teenage boy reported that he often leaves the institution to find quiet places. When asked what he feels or thinks about when he sits in quiet places alone, he said:

*“Nothing, I just listen to music, I feel like I can do what I want to. I can sit as I wish, I cannot be comfortable in the presence of anyone. I cannot do the things I want to when there are many people at home.”*

## Discussion

This is the first qualitative study that explores the personal narratives and experiences of school-age children placed voluntarily by parents in institutions in a country with a strong legacy of a Soviet-style public institutional childcare system. Given that the majority of children in this context were not institutionalized until early-middle childhood and remain in contact with their families throughout, this study provides an opportunity to explore children’s and parents’ reflections on family separation, on upbringing in an institutional environment, and on the psychological consequences of attachment disruption to better understand the consequences of institutionalization for child mental health and well-being.

Unlike the dismal physical conditions in residential institutions during the Soviet era and during the decade of economic crisis after the dissolution of the Soviet Union [48, 49], the living conditions and educational opportunities provided by many institutions in the time following the period of economic growth and investments in the infrastructure of public facilities were decent—indeed, at times they were better than conditions at the study participants’ homes [30]. However, all interviews underlined the emotionally damaging experience that followed family separation and life in the institutional environment for school-aged children.

A lion’s share of the literature on children who have been institutionalized tends to be quantitative in nature, revealing important patterns and trajectories of negative developmental, psychosocial and physical effects. An abundance of literature, spanning multiple contexts, has shown the deleterious developmental consequences of institutional placement, including poor emotional-behavioral regulation, poor physical health, cognitive impairments and stunted physical development [6, 16]. Moreover, extant literature reveals that age at institutional placement and social context (e.g., country of origin) moderate the level and pattern of attachment dysfunction among children who have been institutionalized [50]. This seems consistent with our findings. Yet, while unearthing broadscale patterns might provide important insights about the universal effects of institutional placement, and provide motivation for broader efforts at reform [18], they cannot—by virtue of their methodology—provide insights into the local context, concrete experiences and nuances of child and caregiver’s experiences with institutional placement. This paper adds to the literature by documenting the qualitative accounts of how school-age children and caregivers experience, describe, and interpret the traumatic events associated with the institutional placement. In doing so, this paper adds to the literature by supplementing quantitatively measured mental health or psychiatric symptomatology, to describe the meaning that children and caregivers assign to these experiences and the reasoning behind their behaviors. By providing context-specific, qualitative descriptions of participant experiences, our finding will help to inform mental health practitioners during the therapeutic process.

## Complex developmental trauma

The interviews revealed that the moment of institutional placement—while it was perceived by many parents in this study as a protective act and an investment in their child’s future—was extremely distressing for children and parents. However, parent–child separation was not the first adverse experience faced by children in our study. Narratives from children and parents revealed a history of multiple traumatic experiences that accumulated from early childhood and throughout their institutional placement, from witnessing violence at home to experiencing multiple losses and family instability, as well as living in conditions of severe economic precarity.

The findings reported in this study are most consistent with experiences of complex developmental trauma reported by children in out-of-home placements in sub-Saharan Africa, Australia, India and other parts of the world [51–53]. Complex developmental trauma is characterized by the early life experiences of the multiple, chronic, and prolonged traumatic events that often occur in the context of the caregiving system and lead to wide-ranging and long-lasting adverse outcomes [37].

The toxic stress of the multiple adverse events that these children have experienced may have had a cumulative effect on children’s socioemotional well-being [54], particularly by undermining their emotional and behavioral regulation capacities [55–57]. According to emotion regulation theory [40], experiencing trauma affects the ways in which individuals regulate emotions and communicate with others, causing interpersonal conflict and disjuncture in self-perception. For example, children whose emotional states are not validated or recognized may have difficulty recognizing emotion in others. Children whose emotions were not effectively distracted or soothed by their caregivers may struggle to regulate intense emotions [58]. Thus, institutionalization and parent–child separation may result in emotion regulation difficulties for children with long-lasting impact. Without a constantly available supportive caregiver to respond to children’s emotional cues and to validate emotional experiences, children were left largely to manage their emotions alone, resulting in feelings of sadness, withdrawal and an understandable lack of trust toward their other people, including their own parents.

The disruption of attachment at all stages of childhood and development between the children in this study and their caregivers suggests significant social-emotional difficulties. Two attachment disorders—Disinhibited Social Engagement Disorder and Reactive Attachment Disorder (RAD)—have been commonly observed among children with institutional upbringing and severe deprivation in early childhood [50, 59]. None of the children in this study were observed to present with indiscriminately friendly behavior toward new adults. This is a commonly reported disinhibited attachment disruption pattern in prior studies that focused primarily on children from institutions in the Eastern European and fSU regions who were separated at birth and had no later contact with their biological parents [6, 60]. However, this study did not assess this behavior directly.

## System-induced trauma

In addition to exposure to personal and family-related traumatic events, separation from family, involvement in the child welfare system and placement in closed residential facilities can exacerbate a child’s already undermined functioning [61]. This is often referred to as system-induced trauma: when organized systems of care, while designed to protect children, can create trauma [38].

According to this study, the complex dynamics of institutional placement while maintaining periodic contact with parents substantially affected the attachment security of most children. Children who were placed at an older age (in primary or middle school) and who were able to spend their early childhood with their families more openly expressed their longing for their parents, along with empathy for their parents’ financial precarity and their desires to provide opportunities for their own children. Feelings of loneliness and sadness were more common among these children and were equally observed among boys and girls.

Younger children, however, at times presented as reserved, rejecting, and ‘*cold*’ toward their parents either during visitations or following reunification. Developmentally unable to grasp the context of separation, younger children demonstrated ambivalence and confusion. They struggled to reconcile their understanding of their parents’ financial hardship and their personal feelings of resentment toward their parents due to the perceived injustice they endured following institutionalization, while also yearning for their parents’ presence. These children often seemed to exhibit emotionally detached or unaffected attitudes, some even describing that life in the institution was better than at home. These aforementioned behaviors could be a consequence of disturbances of attachment resulting from parent separation. Children in our sample who presented with emotional detachment from their caregivers tended to detach or stop forming emotional attachments in response to being uncertain about the kind of reaction they would get from their parent or caregiver, especially when the caregiver was not regularly present due to institutional placement. While some behavioral signs may resemble the Reactive Attachment Disorder (RAD), according to the current psychiatric classifications, symptoms should be observed by the age of 5. RAD is one of the most poorly understood and studied psychiatric conditions [62], and more research is warranted with this population to understand attachment disturbances among children who were separated later in life.

## Social exclusion and stigma

Along with managing multiple complex traumas and the stress of separation, children and their mothers may face social and community exclusion after the children’s father left and the children were placed in an institution. Most children interviewed who had a history of institutionalization were the children of divorce whose fathers were completely disengaged following the separation. Within Azerbaijani culture, it is considered a significant stigma for a married couple to divorce and consequently for a child to be raised without a father; divorce and single parenthood are thus highly stigmatized [29]. Prior studies have shown that mothers who placed their child in institutions in Azerbaijan, often as the result of divorce, faced alienation not only from the wider community but also from other family members, leaving them in an economically precarious position with little of the social support that is crucial in collective societies like Azerbaijan [30]. While going through a parental divorce is distressing for children, a social environment condemning divorce is compounded by the dissolution of the nuclear family. For example, in this study, we found that some boys expressed shame at being raised without a father when visiting cousins who were raised by their fathers. Similarly, some mothers expressed that their sons’ challenging behavior was the result of having been raised without a father, suggesting that this stigma may also be held within the family unit itself [30]. Mothers face significant criticism from family members and the community for having been divorced, which within a collectivist culture excludes mothers and their children from a principal form of social and economic support [30].

This study has a number of limitations to note. This is an exploratory study, and the list of described potentially traumatic experiences is not exhaustive. The use of convenience sampling limited our access to particular types of institutions and families. Participants were recruited only from two cities of Azerbaijan and—although it includes institutions from the country’s largest urban area and a smaller town for comparison—its findings may not be generalizable throughout the whole country. Guardian interviews were collected only from mothers and grandmothers as primary caregivers. Fathers’ perceptions have not been described, as none of the sampled children from institutions had fathers as primary caregivers. In the presence of the interviewer, children may underreport the traumatic or emotionally challenging experiences out of fear or lack of trust, and more ambivalent and neutral comments may be overreported.

## Conclusion

Our sample is distinct from samples represented in prior research on child institutionalization in post-Soviet countries, where children tend to be in infancy at the time of institutional placement, have no contact with parents, are more likely to be adopted, and most frequently present with disinhibited or indiscriminately friendly behavior as a result of attachment disruption [20, 63]. The majority of children in Azerbaijan tend to be in middle to late childhood at the time of institutionalization, remain in contact with parent(s) throughout, and are likely to be reunified with their parent(s) before adulthood, which has distinct implications for how disruptions of attachment manifest. Thus, the process of separation in children with a history of family upbringing produced a significantly distinct emotional reaction, as opposed to children who had been placed at birth.

Addressing the complex trauma histories of children and caregivers is crucial to understanding the context of stress during deinstitutionalization and helping families move through the reunification process. While many psychosocial interventions for children leaving institutions focus on social skills and parental behavioral management, they do not typically address the underlying sources of internalizing or externalizing problem behaviors and do not specifically target relational trauma. Moreover, with welfare state devolution, the ongoing privatization of childcare and education, as well as trends toward de-institutionalization, families have become increasingly responsible for the wellbeing of their children [19, 64]. Targeting attachment issues by integrating trauma-informed and family-strengthening approaches could help children and caregivers overcome unresolved conflict and suppressed feelings, strengthen familial bonds, reduce shared stress and social isolation, and buttress a sense of security and trust within family relationships [65]. To address the mental health needs of children, context-specific interventions should target concerns unique to deinstitutionalized children (e.g., readjustment after isolation from society, resentment towards parents, stigma at school or community, and envy towards noninstitutionalized siblings). Current efforts at expanding psychiatric care and community mental health in the region, specifically for children and adolescents, provide a ripe context for expanding trauma-informed care to children who are reuniting with their parents [66]. Strengthening children’s capacities to regulate emotions is crucial in preventing negative mental health outcomes. Programs should also acknowledge the various patterns of child–parent attachment difficulties that children may manifest depending on the age of separation. Following the deinstitutionalization reforms, the subsequent return of children to their families provides an important step toward improving the child’s environment. However, this process may be fraught with challenges if children’s emotional and behavioral difficulties resulting from multiple traumatic experiences are not addressed in a timely and adequate manner.

## Data Availability

Qualitative data analyzed during this current study are not publicly available due to the size of interviews, but are available from the corresponding author on reasonable request with the clear description of study aims.
